# Study on the experimental performance by electrolysis-integrated ecological floating bed for nitrogen and phosphorus removal in eutrophic water

**DOI:** 10.1038/s41598-020-64499-y

**Published:** 2020-05-06

**Authors:** Cheng Yan, Mingxuan Wang, Tangming Ma, Shunqing Yang, Ming Kong, Jianing Shen, Liuyan Yang, Yan Gao

**Affiliations:** 1 State Key Laboratory of Pollution Control and Resource Reuse, School of the Environment, Nanjing University, Nanjing, 210023 P.R. China; 2 Nanjing Institute of Environmental Sciences, Ministry of Ecology and Environment, Nanjing, 210042 P.R. China

**Keywords:** Freshwater ecology, Pollution remediation

## Abstract

The new-type electrolysis-integrated ecological floating beds (EEFBs) were set up to study their water removal ability due to the excellent water treatment capacity of electrolysis, this enhanced EEFBs were made of polyethylene filled with biochar substrate and in middle of the substrate placed the Mg-Al alloy served as anode and graphite served as cathode. The results show that removal rates of total nitrogen (TN), ammonia nitrogen (NH_3_-N), total phosphorus (TP) and phosphate (PO_4_^3−^-P) by the EEFBs increased 53.1%, 96.5%, 76.5% and 74.5%, respectively. The electrolysis reaction was the main pathway for TN and TP removals in the EEFBs. A higher concentration of hydrogen autotrophic denitrification bacteria was recorded in the substrate of the EEFBs than that in the traditional ecological floating beds (EFBs) (*p* < 0.05), suggesting that the electrolysis may have enhanced the NO_3_^−^-N removal efficiency of the EEFBs by promoting the growth and reproduce of hydrogen autotrophic denitrification bacteria. The *in-situ* formation of Mg^2+^ and Al^3+^ ions from a sacrificial Mg-Al alloy anode, caused PO_4_^3−^-P and other suspended matter flocculation, improved phosphorus removal and simultaneously reduced turbidity. Thus, electrolysis-integrated ecological floating bed has high nitrogen and phosphorus removal potential in eutrophic water.

## Introduction

Eutrophication has become a serious matter of concern in aquatic ecological research, widespread occurrence of water eutrophication results in the loss of ecological integrity, a decreased aquatic biodiversity, the disappearance of submerged vegetation, and the potential production of toxins^1^. Diffuse nitrogen (N) and phosphorus (P) pollution are currently the main drivers of eutrophication^2^. Ecological restoration technology, such as ecological floating beds (EFBs) have the unique advantage of occupying no land area, operate at low cost, and require simple maintenance; therefore, the EFBs have been widely used as an *in-situ* ecological remediation technology for treating surface water^3,4^. However, problems of seasonal and anaerobic environment constraints on macrophytes and microbes growth, the limited standing biomass^5–7^, decrease of adsorption capacity of substrate^7–11^ and the larger cover area^4^ usually restrict the usage of EFBs. Thus, the enhanced EFBs which have a long term stable removal capacity needed to be researched.

When used, electrolysis in eutrophic water bodies can simultaneously promote heterotrophic denitrifier’ growth and reproduce to enhance NO_3_^−^-N removal^12–15^ and to enhance PO_4_^3−^-P removal through sacrificial anode which can be used as the source of the coagulating ions^16,17^. Some researchers were already using electrolysis reaction in constructed wetlands (CWs) and biofilter to intensify N and P removal since the anode was Fe or Al^18–22^. When the anode was Fe which could produce Fe^2+^ and Fe^3+^ ions during the electrolysis process and Fe(III) oxide generated as the end result. Fe(III) oxide has a reddish-brown color and is often called rust which affected the water transparency when there was too much rust^23^. When used Al as anode provided a higher P removal efficiency as Al anode had a lesser metal-to-P ratio compared to Fe anode, but more Al^3+^ ions has various biotoxicity effects on aquatic organisms such as algae, fish and even humans^24–26^. Excepting Fe and Al, magnesium cations by corrosion and/or electrolytic dissolution were also used to remove P because they have little effect on the aquatic ecosystem^27^. In this study, an enhanced EEFBs were designed by introducing the Mg-Al alloy as the anode, a graphite electrode as the cathode, and biochar as the substrate to explore the nutrient removal ability in the EEFBs. The indoor experiments were carried out: (1) to analyze the N and P removal capabilities of the EEFBs during simulation of eutrophic wastewater; (2) to evaluate the effect of electrolysis on substrate microorganism; and (3) to evaluate the power consumption of EEFBs to enhance N and P removal.

## Results

### Nitrogen and phosphorus removal efficacy

**Figure 1:**
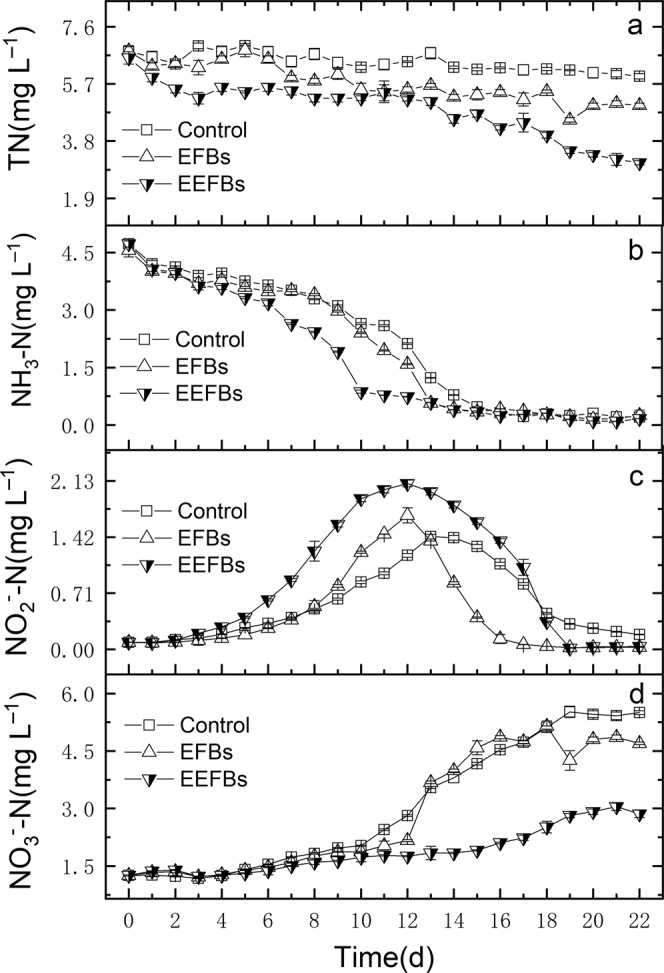
The concentrations of TN (**a**), NH_3_-N (**b**), NO_2_^−^-N (**c**), and NO_3_^−^-N (**d**) in the EEFBs, the EFBs and the control during the purification of eutrophic water, data points represent sample means ± the standard error of the mean.

**Figure 2:**
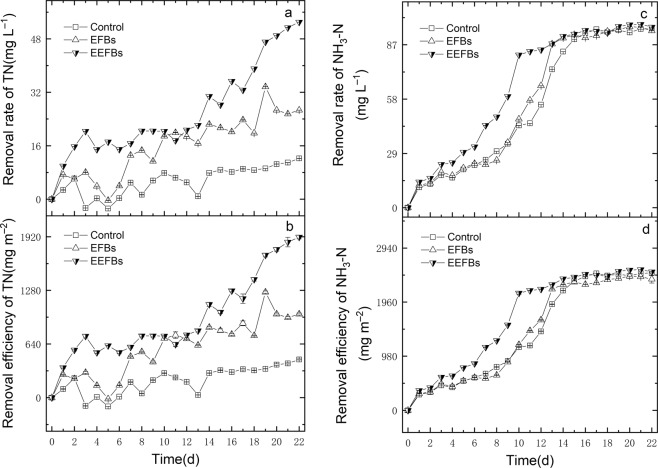
The removal rates and removal efficiencies of TN (**a,b**) and NH_3_-N (**c,d**) in the EEFBs and the EFBs during the purification of eutrophic water, data points represent sample means ± the standard error of the mean.

**Figure 3:**
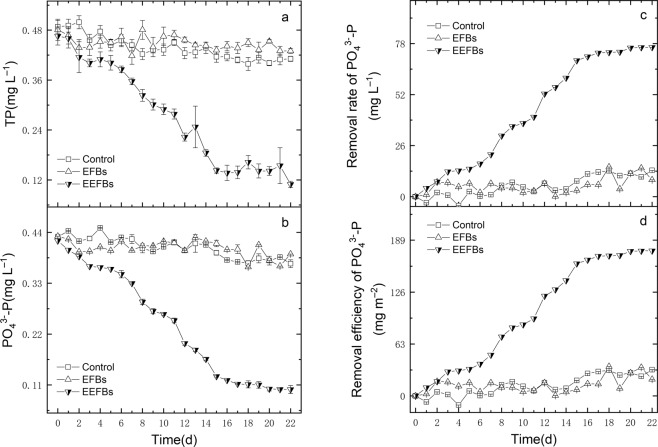
The concentrations of the TP (**a**) and PO_4_^3−^-P (**b**), removal rates of PO_4_^3−^-P (**c**) and removal efficiency of PO_4_^3−^-P (**d**) during the purification of eutrophic water, data points represent sample means ± the standard error of the mean.

At the end of the experiment, the concentrations of total nitrogen (TN), ammonia nitrogen (NH_3_-N), Nitrate nitrogen (NO_3_^−^-N), total phosphate (TP) and phosphate (PO_4_^3^^−^-P) in the EEFBs were lower than those in the EFBs (Figs. 1–3), the EEFBs removed almost 53.1% ± 1.13% of the TN with a concentration of up to 6.56 ± 0.20 mg L^−1^, and the net removal rate of EEFBs and EFBs were 40.9% ± 1.13% and 14.5% ± 1.19%, which represent a markedly significant difference in the TN removal (*p* = 0.006). The removal efficiency of the EEFBs was as high as 1916.58 ± 21.73 mg m^−2^ and was about 1.92 times and 4.20 times higher than the EFBs and the control. For NH_3_-N, the EEFBs, the EFBs and the control showed a dramatic decrease from the mean initial concentration of 4.67 ± 0.1 mg L^−1^ to the final concentrations of 0.21 ± 0.00 mg L^−1^, 0.25 ± 0.14 mg L^−1^ and 0.17 ± 0.00 mg L^−1^ in the water column, respectively (Fig. 1b). The NH_3_-N removal rate in the EEFBs and EFBs were as high as 96.5% ± 0.03% and 94.5% ± 3.00% (Table 1), No significant difference was found between the removal rate of NH_3_-N in the EEFBs and EFBs (*p* = 0.291).

**Table 1:** Removal rate and net removal rate of TN, NH_3_-N, TP and PO_4_^3−^-P by different treatments. Data are means followed by standard errors (± SE) (n = 3). Different letters indicate significantly different removal among the three treatments at *p* < 0.05.

The treatment	TN		NH_3_^−^-N		TP		PO_4_^3−^-P	
	Removal rate(%)	Net removal rate(%)	Removal rate(%)	Net removal rate(%)	Removal rate(%)	Net removal rate(%)	Removal rate(%)	Net removal rate(%)
Control	12.2 ± 1.28a	—	95.6 ± 0.04a	—	15.8 ± 1.26a	—	13.6 ± 1.93a	—
EFBs	26.7 ± 1.19b	14.5 ± 1.19a	94.5 ± 3.00a	—	10.54 ± 1.21b	—	8.9 ± 0.01b	—
EEFBs	53.1 ± 1.13c	40.9 ± 1.13b	96.5 ± 0.03a	0.9 ±0.03a	76.5 ± 1.59c	60.7 ± 1.59a	74.5 ± 2.09c	60.9 ± 2.09a

From 4 to 18 days, the mean NO_2_^−^-N concentrations increased to 1.27 ± 0.66 mg L^−1^, 0.64 ± 0.56 mg L^−1^ and 0.77 ± 0.45 mg L^−1^ in the EEFBs, the EFBs and the control, respectively, the NO_2_^−^-N concentration in the EEFBs was significantly higher than that in the EFBs (Fig. 1c) (*p* = 0.007), and there was no difference in the NO_2_^−^-N concentration in the EFBs and the control (*p* = 0.537). The NO_3_^−^-N concentrations were 2.86 ± 0.09 mg L^−1^, 4.70 ± 0.03 mg L^−1^ and 5.50 ± 0.05 mg L^−1^ in the EEFBs, EFBs and control, respectively at the end of the experiment (Fig. 1d). Electrolysis could enhance NO_3_^−^-N removal in the EEFBs. Compared to the control, there was about 2646.4 mg NO_3_^−^-N were removed by the EEFBs, which was 7.46 times higher than that by the EFBs (354.7 mg), and there was no significant difference between the EFBs and the control (*p* = 0.748).

The initial concentration of TP was about 0.48 ± 0.02 mg L^−1^ and the PO_4_^3−^-P concentration accounted for 89.5% of TP in the experimental eutrophic water. At the end of the experiment, the final TP concentration was reduced to 0.10 ± 0.01 mg L^−1^, and the removal rate was 76.5% ± 1.59% in the EEFBs, which was significantly higher than that of the EFBs (10.54% ± 1.21%) (*p* < 0.001) (Fig. 3 and Table 1). Moreover, the TP net removal rate in the EEFBs was 76.5% ± 1.59% and the PO_4_^3−^-P removal rate was 60.7% ± 1.59%, which was much higher than that of the EFBs.

**Figure 4:**
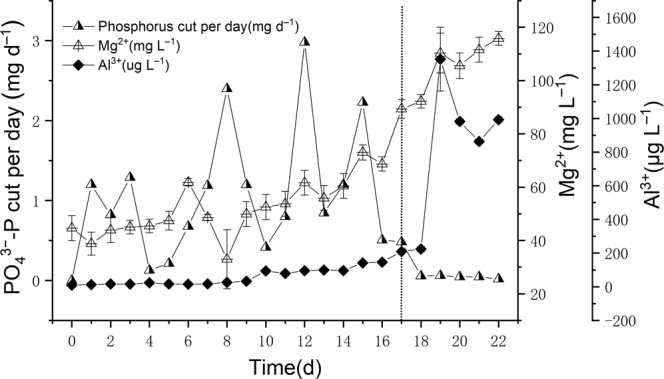
The PO_4_^3−^-P reduction amount per day and Mg^2+^, Al^3+^ ions concentrations in the purification of eutrophic water by EEFBs, data points represent sample means ± the standard error of the mean.

The higher PO_4_^3−^-P removal ability was attributed to the electrolysis reaction and Mg^2+^ and Al^3+^ ions produced by Mg-Al alloy anode. However, PO_4_^3−^-P removal efficiency and Mg^2+^ and Al^3+^ ions concentrations did not always increase indefinitely, the PO_4_^3−^-P reduction amount per day was reduced to only 0.02 mg even the concentrations of Mg^2+^ and Al^3+^ ions were 115.84 ± 2.57 mg L^−1^ and 992.77 ± 16.74 μg L^−1^, respectively in the end of the experiment (Fig. 4).

### Effect of electrolysis on substrate biofilm

**Figure 5:**
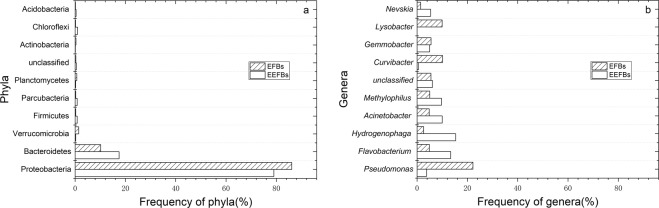
Distribution of bacterial phyla (**a**) and genera (**b**) in the biochar substrate of the EEFBs and the EFBs.

Electrolysis affected the bacterial community richness of biochar substrate, because the operational  taxonomic  unit (OTU) number (*p* = 0.005) and ACE-index (*p* = 0.008) as well as the Chao1-index (*p* = 0.008) were significantly different between the EEFBs and EFBs (Supplementary Table S1) which mean some microbial species were enriched in the EEFBs. No significant difference was found in the diversity of the microbial community distribution between EEFBs and EFBs since the differences between the Shannon index (*p* = 0.519) and Simpson index (*p* = 0.883) were small. The bacterial phyla in biofilm on the EEFBs substrate mainly include Proteobacteria (78.99%), Bacteroidetes (17.48%), Firmicutes (0.84%), Parcubacteria (0.83%), Actinobacteria (0.11%), Verrucomicrobia (0.27%) and Nitrospirae (0.04%). Comparing to EFBs, the proportion of Bacteroidetes, Firmicutes and Nitrospirae increased while the Proteobacteria decreased (Fig. 5a).

**Figure 6:**
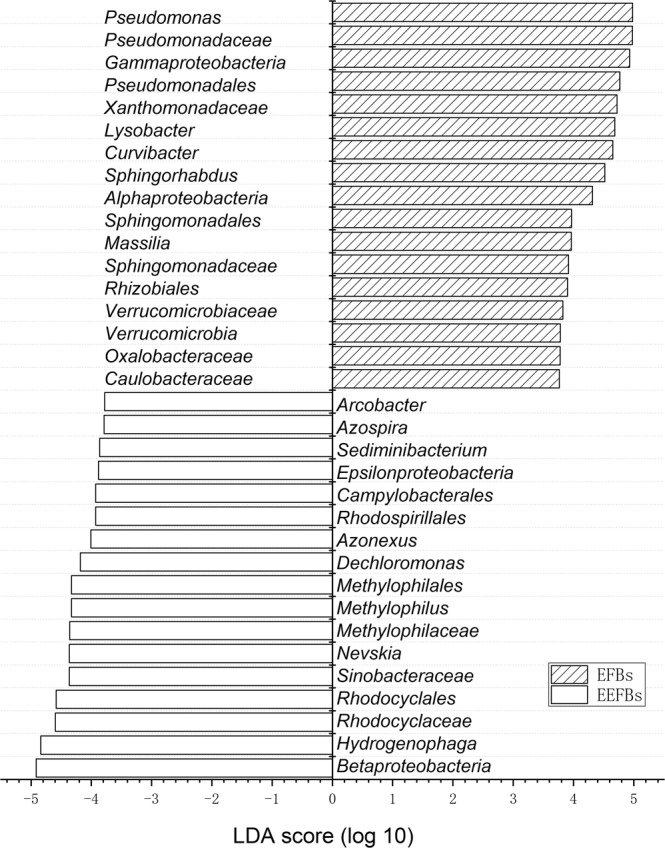
Analysis of taxonomic relative abundances using LEfSe analysis showed the main function of a bacterial community enriched in the substrate biofilm of the EEFBs during the electrolysis process. Note: This histogram shows a significantly enriched bacterial taxa among groups based on the linear discriminant analysis (LDA) score.

The genera of bacteria in the biochar substrate biofilm of the EEFBs changed as the functional group was changed and multiple microbial groups were enriched. From Fig. 5b, we concluded that the genera of *Hydrogenophaga, Flavobactrium, Pelomonas, Thiobacillus, Simplicispira, Zoogloea, Optitutus, Pseudomonas, Methylophilus*, and *Thauera* were identified as the main denitrifying bacteria in the biochar substrate biofilm of EEFBs. The addition of electrolysis in the EFBs could effectively increase the bacterial content of *Hydrogenophaga* (15.33%) and *Flavobactrium* (13.35%). Moreover, *Dechloromonas* (2.96%) was enriched in the biochar substrate, as *Pseudomonas*, *Curvibacter*, and *Lysobacter* species decreased. *Hydrogenophaga* was the first abundant dominant genus in the biochar substrate of the EEFBs and plays a vital role toward removing NO_3_^−^-N (Figs. 5b and 6). Most of the bacteria in these genera have proved capable of denitrification and can use H_2_ as electronic donor for growth and reproduction. Electrolysis can produce a large amount of H_2_, which increased the water pH to a certain extent (Supplementary Fig. S1).

### Electrolysis power consumption for nutrient removal

**Figure 7:**
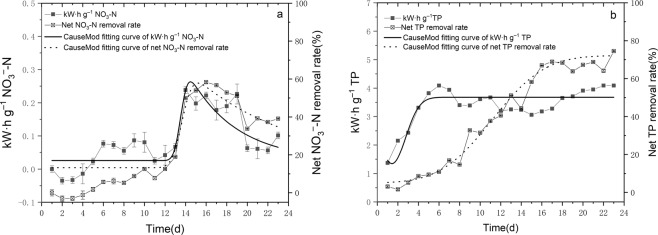
The electrolysis power consumption and net removal rates of TN (**a**) and TP (**b**) by the EEFBs during the purification of eutrophic water.

Since electricity was needed during the electrolysis process, the power consumption in the N and P removal processes also needed to be analyzed and compared. From Fig. 7a,b, we found that electrolysis significantly promoted the net removal of NO_3_^−^-N and TP, and the energy consumption by NO_3_^−^-N removal was lower than that of TP (*p* < 0.001). From the graph and GaussMod fitting curve, the trends of the net NO_3_^−^-N removal rate and energy consumption were more consistent. After the twelfth day, the net NO_3_^−^-N removal rate increased sharply to 60%, which could be attributed to the enhanced NH_3_-N oxidation which results a higher substrate concentration of NO_3_^−^-N and the high growth of hydrogen autotrophic bacteria which promoted the denitrification.

The electrical energy used in the TP removal also had an obvious correlation with its net removal rate. From Fig. 7b, the energy consumption used TP removal in the EEFBs increased sharply from 1.37 ± 0.02 kW·h g^−1^ to 4.09 ± 0.01 kW·h g^−1^ as the net TP removal rate increased from 3.08% ± 0.33% to 10.90% ± 0.27%, and the energy consumption was basically stable after the sixth day. From the CauseMod fitting curve of energy consumption and net TP removal rate, we found that compared with electrical energy, a time lag occurred in the net TP removal rate.

## Discussion

Electrolysis was reported as an potential alternative to traditional constructed wetland and biofilter for remediation of N and P rich water with the direct and indirect functions of cathode and anode^19–21^. In our experiment, the electrolysis combined with EFB added biochar substrate and vegetated with *Iris sibirica* L. was effectively remove TN and TP in the water column with removal rates of 53.1% and 76.8% which were higher than that of EFBs, and the removal efficiencies of TN (1916.58 mg m^−2^ day^−1^) (*p* = 0.009) and TP (176.13 mg m^−2^ day^−1^) were significantly higher than that of EFBs. The previous study found that the floating treatment wetland aided remediation of TN (128 mg m^−2^ day^−1^) and PO_4_^3^^–^-P (10.6 mg m^−2^ day^−1^) as the EEFBs greatly increased the removal rates of N and P^28^. NH_3_-N in the water column can be converted into NO_2_^−^-N and NO_3_^−^-N under aerobic conditions by nitrification bacteria (NB), and then the NO_2_^−^-N and NO_3_^−^-N are transformed into N_2_ or N_2_O by denitrification bacteria (DB) under the condition of anoxia and escape from the water body^29,30^. Adding the electrolysis in EEFBs may have enhanced the nitrification and denitrification process, as the intermediate product of mean NO_2_^−^-N concentration was significantly higher than that in the EFBs (*p* = 0.035), especially in 8 to 17 days the mean concentration was as high as 1.66 mg L^−1^ which was 2 times than that in the EFBs (0.825 mg L^−1^) (Fig. 1). As NO_2_^−^-N was the main intermediate product of nitrification and denitrification which indicated those processes were strong in the EEFBs than that in EFBs (*p* = 0.004). Along with the experiment process, NH_3_-N was transformed by NB to NO_2_^−^-N and NO_3_^−^-N, which enhanced the growth of DB and process of denitrification in the EEFBs and the EFBs. The NO_3_^−^-N concentration decreased significantly (*p* < 0.001) than that in the EFBs from 12 to 20 days since the cathode produced H_2_ which were electron donors for hydrogen autotrophic denitrification bacteria to breed and reproduce, this favored the removal of NO_3_^−^-N and other nutrients^12,31,32^. The results showed that the contents of *Hydrogenophaga* (15.33%), *Flavobactrium* (13.35%) and *Dechloromonas* (2.96%) were enriched on the substrate of the EEFBs, higher total amount of hydrogen autotrophic denitrification bacteria were recorded in the EEFBs than that in the EFBs (Figs. 5 and 6), as more studies have found that these three types of bacteria are enriched in the hydrogen autotrophic denitrification system^33,34^. Thus, adding electrolysis to the ecological floating bed affected the DB composition and diversity of the bacterial community on the substrate as an efficient NO_3_^−^-N removal was realized.

The main P purification pathway of traditional EFB was adsorption of adding various substrates such as zeolite, sponge iron, rice straw, plastic filling and ceramsite. Because the adsorption of the substrate decreased the P removal rate was reduced in the long run^35,36^, the longer the operation time was, the second release of P would occur and the PO_4_^3−^-P removal was negative in effluent^28^. The EEFBs effectively removed TP and PO_4_^3−^-P in the water column over a long term sustainability, with high removal rates of 76.5% and 74.5%, respectively in our study (Figs. 4 and 5), and the removal efficiencies of TP and PO_4_^3−^-P (*p* < 0.001) were significantly higher than that in EFBs; the special electrolysis in the EEFBs adequately removed P in the water column which was caused by released Mg^2+^ and Al^3+^ ions during the electrolyzing process as Mg-Al alloy was anode and this enhanced pathway was not affected in the long run^37^. Thus, the EEFBs can be used to enhance the removals of N and P in winter, especially when the dissolved oxygen (DO) in water body are relatively low such as highly polluted river in city.

From the previous study, we could conclude that apart from the role of substrate, plants uptake and microbes played a major role in traditional EFB for the removals of N and P from eutrophic water, but the absorption of plants and microbes are mainly restricted by low temperature in winter, and the plant corruption can also lead to the release of nutrients such as N and P, so the need for whole-plants harvesting and management also need to be considered in the long run^9,11,38,39^.

In our present study, *I. sibirica* had a higher fresh weight, plant height and root length in the EFBs than that in the EEFBs, since the absolute growth rate (ARG) in the EEFBs was 10.82 ± 12.54 mg d^−1^, which was significantly lower than that in EFBs as the ARG was 101.71 ± 25.65 mg d^−1^ (*p* < 0.05) (Supplementary Table 2). Its revealed that the growth of *I. sibirica* may was affected by the reduced nutrients or the reaction of electrolysis in eutrophic water during the entire experiment time, comparing to EFBs, about 26.4% TN and 65.9% TP were directly or indirectly removed by the introduction of electrolysis in the EEFBs (Table 1). And with increasing the processing time, more pollutants in water were flocculated and precipitated by Mg^2+^ and Al^3+^ ions produced by electrolysis of the Mg-Al alloy anode lead to the average value of turbidity (1.11 ± 0.20 NTU) decreased about 50.45% comparing with the EFBs (Supplementary Fig. S1). The contribution of electrolysis was the main cause of higher purification efficacy in the EEFBs. Thus, the EEFB is a new type of EFB which can overcome the low efficiency of traditional floating bed for N and P removals through electrolysis reaction, and the nutrients mainly precipitated in the sediment which suggested whole sediment management. Therefore, the sediment management should be considered in the further research of nutrient treatment especially for P.

## Conclusions

In this study, a new type of electrolysis combined with ecological floating bed was designed and applied to control N and P pollution in eutrophic water. The results showed that the net removal rates of N and P were significantly better than those rates representing traditional ecological floating beds (*p* < 0.05), especially for removals of TN, NO_3_^−^-N and TP. The electrolysis process in the EEFBs can also produce H_2_ by anodes for hydrogen autotrophic denitrification bacteria such as the genera *of  Hydrogenophaga*, *Flavobacterium* and *Dechloromonas* in the biochar substrate biofilm were enriched and improved the removal efficiency of NO_3_^−^-N. A stable high P removal rate was obtained by releasing Mg^2+^ and Al^3+^ ions during the electrolyzing process to enhance that eutrophic water’s P flocculation capacity. The ARG of *I. sibirica* was 10.82 ± 12.54 mg d^−1^, which was significantly lower than that growth in the EFBs as the ARG was 101.71 ± 25.65 mg d^−1^ (*p* < 0.05), which infers that electrolysis influenced the growth of the plants and the plants uptake rate was lower than the EFBs. Compared with electrical energy process there was a time lag in the net TN and TP removal rates and the P removal rate was not proportional with the energy at a later stage, which needed additional energy to optimize the TN and TP removal efficacies by electrolysis.

## Methods

### Electrolysis ecological floating bed design and eutrophic water

**Figure 8:**
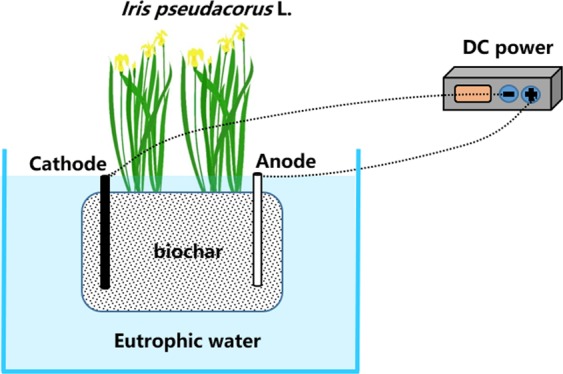
Schematic diagram of electrolysis-ecological floating bed (EEFB).

In this research, the EEFBs and the traditional EFBs both had the same surface area of 1,089 m^2^ and were designed to compare their removal efficiencies in eutrophic waters under similar conditions. The EEFBs were made of polystyrene board 33 cm wide × 33 cm long × 5.5 cm thick. A 15 cm diameter planting hole was at the center of the board. Two nylon bags of biochar substrates were hung on the two sides of the 15-cm inner square frame (15 cm wide) of the EEFBs (Fig. 8). Each bag of the biochar substrates weighed 1.2 kilograms. The biochar had a diameter ranging from 1 mm to 3 mm and was made from bamboo poles (*Phyllostachys heterocycla* (Carr.) Mitford cv. *Pubescens*), which were fired under a controlled oxygen condition for three days, then fired at 800 °C for 48 h, and then allowed to cool naturally to ambient temperature. Before use, biochar was immersed in 1 mol L^−1^ hydrochloric acid for two hours then rinsed with deionized water until neutral. Later, they were dried before use. The anodes and cathodes were both set in the middle of the bag. The anodes were the Mg-Al alloy with an atomic percentage of 72.52% Mg. The cathodes were graphite. Each anode and cathode was 250 mm long, 150 mm wide, and 0.2 mm thick. A 5% hydrochloric acid was used to activate the electrodes for 15 mins before use; They were then rinsed with deionized water. The distance between cathodes and anodes was 120 mm. The effective working area of the electrodes was 135.41 cm^2^. The electrodes were connected using copper wires with a diameter of 1 mm to a Model KXN-3020D DC regulated power supply (Zhaoxin Electronic Instruments and Equipment Co., Ltd., Shenzhen, China) with a voltage ranging between 0 V and 30 V and an amperage between 0 A and 5 A. This provided a constant current for electrolysis with a current density of 0.37 mA cm^−2^. The *I. sibirica* seedlings were cultivated for 10 days prior to the experiment with a similar height of 30.7 cm and fresh weight of about 5.54 g. These ornamental macrophytes were planted in the hole of the EEFBs and the EFBs.

The EEFBs and the EFBs were both placed in a polyethylene plastic box with a total volume of 100 L. The box with only the experimental eutrophic water served as the control. With eutrophic waters of 60 L taken from a river near Nanjing city, the concentrations of TN, NH_3_-N, NO_3_^−^-N, NO_2_^−^-N, TP, PO_4_^3−^-P, COD in the experimental water were 6.78 ± 0.2 mg L^−1^, 4.72 ± 0.17 mg L^−1^, 1.26 ± 0.02 mg L^−1^, 0.09 ± 0.01 mg L^−1^, 0.48 ± 0.03 mg L^−1^, 0.43 ± 0.01 mg L^−1^ and 48 ± 7 mg L^−1^, respectively. The parameters of DO, ORP (oxidation reduction potential) and pH were 6.44 ± 0.14 mg L^−1^, 122.9 ± 4.2 mV and 8.03 ± 0.03, respectively. During the experiment, three replicates wastewater samples were collected every 24 hours to determine nutrient values and physical and chemical indicators of water quality. The experimental apparatuses were maintained within a temperature range of 25 °C and 30°C. During the period of the experiment, the water loss from evaporation and transpiration was supplied by adding deionized water to the original level every two or three days.

### Sampling of water, biochar substrate, and plants

Water samples were collected from the reactor every 24 hours to determine the physical and chemical indicators of water quality. The pH value, temperature, ORP values and turbidity, total dissolved solids (TDS), and salinity were immediately measured using a multi-parameter water quality YSI ProPlus (YSI Inc., Yellow Springs, OH, USA) at a depth of 0.5 m beneath the water surface. pH and DO were immediately measured by a portable Hach HQ30d multi-parameter analyzer, a PHC101-30 pH electrode, and a LDO101-03 DO electrode (all from Hach Company, Loveland, CO, USA). NO_3_^−^-N (dihydrochloride), NO_2_^−^-N (N-(1-naphthyl) ethylenediamine dihydrochloride), NH_3_-N (natrium reagent), TN (alkaline potassium persulfate digestion) and PO_4_^3−^-P (ascorbic acid method) were measured using an ultraviolet and visible (UV-Vis) V1800 spectrophotometer (Shimadzu Corp., Kyoto, Japan) after the corresponding standard pretreatment and reagent addition were completed. All the testing procedures were according to Standard Methods for the Examination of Water and Wastewater by the Ministry of Ecology and Environment of the People’s Republic of China^40^.

To quantify the *I. sibirica* growth in the EEFBs and the EFBs, three plants of similar height and weight were compared to the seedlings planted in the floating beds and tested when the experiment was initiated. After washing with deionized water and drying with absorbent paper, the plants were weighed on an electronic scale to determine their total fresh weight, plant height, root length and branch number and AGR^41^. At the end of the experiment, the plants in the EEFBs and the EFBs were collected, and the same parameters were recorded using the same methods.

### Characterization of the microbial community and diversity analysis

The bacterial community was examined in the biofilm on biochar of the EEFBs and the EFBs, using DNA-based molecular techniques. About 250 g of biochar were taken from each floating bed when the experiment was ended. At that time, phosphate buffer saline (PBS) solution (containing 9.3 g L^−1^ of K_2_HPO_4_ and 1.8 g L^−1^ KH_2_PO_4_) was added and washed into the liquid at 50 kHz for 30 mins; then, the washed liquid was centrifuged at 1200 rpm for 2 mins, and the precipitation was extracted according to the instructions of OMEGA E.Z.N.ATM-Bind Soil DNA Kit. High-throughput gene sequencing was used to identify and estimate changes in the relative abundances of bacteria.

DNA concentration and purity were determined by a NanoDrop ND-1000 microspectrophotometer (NanoDrop Technologies, Wilmington, DE, USA). Three replicate DNA extractions were combined into one sample for Illumina high-throughput sequencing (HTS) after genomic DNA was extracted. HTS was performed externally (by Sangon Biotech, Shanghai, China) using standard protocols on a MiSeq platform (Illumina, Inc., San Diego, CA, USA). The resulting sequencing data were processed using QIIME v 1.8.1^42^. Archaeal, bacterial, and eukaryotic reads were distinguished based on differences in their PCR primers and then analyzed separately. The reads were then clustered into operational OTUs based on 97% similarity with UCLUST^43^. Representative sequences from each OTU were assigned a taxonomy using an RDP Classifier^44^, with a minimum support threshold of 80%, and aligned using the Greengenes reference database (version 13_8)^45^ by PyNAST^42^. Raw sequence data (FASTQ files) generated for this study have been deposited in NCBI’s Sequence Read Archive under BioProject number 16S190230. Microbial diversity was measured by analyzing the alpha and beta diversities, as determined using the QIIME pipeline and based on the OTUs. For alpha diversity, Chao1 richness and the Shannon and Simpson diversity indices were calculated using QIIME^45^.

### Data analysis

All data analyses were performed in triplicate, and the data were expressed as mean ± standard errors. To evaluate the removal performance of the EEFBs, the removal efficiency (L, mg m^−2^), net removal rate (R_n_, %), energy consumption (W, k·Wh g^−1^), and AGR (mg d^−1^) of plants were analyzed, according to the following formulas.

1 $${\rm{L}}=({{\rm{C}}}_{0}{{\rm{V}}}_{0}-{{\rm{C}}}_{{\rm{i}}}{{\rm{V}}}_{{\rm{i}}})/{\rm{A}}$$

2 $${{\rm{R}}}_{{\rm{n}}}={{\rm{R}}}_{{\rm{t}}}\mbox{--}{{\rm{R}}}_{{\rm{c}}}$$

3 $${{\rm{W}}}_{{\rm{i}}}=({\rm{UIt}}\times 1000)/({{\rm{C}}}_{{\rm{t}}}-{{\rm{C}}}_{0}){\rm{V}}$$

4 $${\rm{AGR}}=({{\rm{W}}}_{1}-{{\rm{W}}}_{2})/({{\rm{t}}}_{2}-{{\rm{t}}}_{1})$$

where L (%) represents the removal efficiency of different pollutants; C_0_ (mg L^−1^) represents the initial concentration of pollutants; V_0_ (L) represents the volume of eutrophic water; Ci and Vi refer to the pollutant concentration (mg L^−1^), and the water volume (L) was measured in day i. A (m^2^) is the area of each floating bed; R_n_ (%) represents the net removal rate of each treatment; R_t_ (%) represents the total ultimate average removal rate in each treatment; R_c_ (%) represents the average removal rate of the control; Wi (k·Wh g^−1^) represents the energy consumption of the electrolysis reaction in the EEFBs; U (V) represents the voltage between electrodes; I (mA) represents the current passing through the electrodes; C_0_ (mg L^−1^) represents the initial concentration of pollutants, C_t_ (mg L^−1^) is the concentration of pollutants in day i, V (L) is the volume of eutrophic water in each treatment; t (d) represents the reaction time; W_1_ (mg) and W_2_ (mg) refer to the initial and final fresh weights of the plants, respectively; and t_1_ (d) and t_2_ (d) represent the initial and final experimental time (days), respectively.

The concentrations (mg L^−1^) of N and P, removal rate (%), the removal efficiency (L, mg m^−2^) and energy consumption (W, k·Wh g^−1^) in the EEFBs, the EFBs and control group were performed with SPSS 23.0 (SPSS Inc., Chicago, IL, USA), the significant differences of energy consumption (W, k·Wh g^−1^) between treatments were determined by an analysis of variance (ANOVA) followed by post-hoc testing using Tukey’s HSD test. A *p* value of less than 0.05 was used to determine if the differences were statistically significant use one way ANOVA by Microsoft excel 2016.

## Supplementary information

Supplementary Information.

Supplementary Information2.
