# Supplementary material for: Study on the experimental performance by electrolysis-integrated ecological floating bed for nitrogen and phosphorus removal in eutrophic water
Source: Sci Rep. 2020 May 6;10:7619. doi: 10.1038/s41598-020-64499-y (PMC7203143; doi:10.1038/s41598-020-64499-y)
Supplement: Supplementary file 1 — Supplementary Information. [file 41598_2020_64499_MOESM1_ESM.docx]

**Study on the experimental performance by electrolysis-integrated ecological floating bed for nitrogen and phosphorus removal in eutrophic water**

Cheng Yan^1^, Mingxuan Wang^1^, Tangming Ma^1^, Shunqing Yang^1^, Ming Kong^2^, Jianing Shen^1^, Liuyan Yang^1^, Yan Gao^1*^

^1^State Key Laboratory of Pollution Control and Resource Reuse, School of the Environment, Nanjing University, Nanjing, 210023, P. R. China.

^2^Nanjing Institute of Environmental Sciences, Ministry of Ecology and Environment, Nanjing, 210042, P. R. China.

**Supplementary Figure**

Figure S1. The main water quality variables of ORP (a), DO (b), turbidity (c), TDS (d), salinity (e), conductivity (f), pH (g), and temperature (h) in the EEFBs, the EFBs and the control during the purification of eutrophic water
