# Supplementary material for: Study on the experimental performance by electrolysis-integrated ecological floating bed for nitrogen and phosphorus removal in eutrophic water
Source: Sci Rep. 2020 May 6;10:7619. doi: 10.1038/s41598-020-64499-y (PMC7203143; doi:10.1038/s41598-020-64499-y)
Supplement: Supplementary file 2 — Supplementary Information2. [file 41598_2020_64499_MOESM2_ESM.docx]

**Study on the experimental performance by electrolysis-integrated ecological floating bed for nitrogen and phosphorus removal in eutrophic water**

Cheng Yan^1^, Mingxuan Wang^1^, Tangming Ma^1^, Shunqing Yang^1^, Ming Kong^2^, Jianing Shen^1^, Liuyan Yang^1^, Yan Gao^1*^

^1^State Key Laboratory of Pollution Control and Resource Reuse, School of the Environment, Nanjing University, Nanjing, 210023, P. R. China.

^2^Nanjing Institute of Environmental Sciences, Ministry of Ecology and Environment, Nanjing, 210042, P. R. China.

**Supplementary Tables**

Table S1. The Analysis of bacterial diversity index in substrate of the EEFBs and the EFBs

| **Sample** | **OTU** | **Shannon** | **ACE** | **Chao1** | **Simpson** |
| --- | --- | --- | --- | --- | --- |
| EEFBs | 723 ± 88.43 a | 3.89 ± 0.45 a | 810.75 ± 103.27 a | 815.32 ± 92.28 a | 0.06 ± 0.03 a |
| EFBs | 1082.33 ± 70.47 b | 4.08 ± 0.10 a | 1203.33 ± 97.90 b | 1184.96 ± 88.91 b | 0.07 ± 0.02 a |

Table S2. The growth characteristics of *Iris sibirica* L. in the EEFBs and EFBs. Data are means followed by standard errors (± SE) (n = 3). AGR: absolute growth rate. Different letters indicate significantly different among three treatments at *p* < 0.05.

| **Time** | **The treatment** | **Total fresh weight(g)** | **Plant height(cm)** | **Root length(cm)** | **Branch number** | **AGR(mg d^–1^)** |
| --- | --- | --- | --- | --- | --- | --- |
| Before experiment | EFBs | 4.69 ± 0.90 | 26.37 ± 2.57 | 8.23 ± 0.15 | 5.33 ± 0.58 | ‒ |
|  | EEFBs | 6.10 ± 1.62 | 34.23 ± 2.91 | 6.73 ± 1.15 | 5.67 ± 0.58 | ‒ |
| After experiment | EFBs | 6.73 ± 1.42a | 38.5 ± 4.35 a | 11.8 ± 3.81a | 7.00 ± 1.00a | 101.71 ± 25.65a |
|  | EEFBs | 6.32 ± 1.87a | 30.47 ± 5.34a | 7.80 ± 0.61a | 7.67 ± 0.58a | 10.82 ± 12.54 b |
